# An Extended Twin-Pedigree Study of Different Classes of Voluntary Exercise Behavior

**DOI:** 10.1007/s10519-019-09990-7

**Published:** 2020-01-23

**Authors:** Matthijs D. van der Zee, Q. Helmer, D. I. Boomsma, C. V. Dolan, E. J. C. de Geus

**Affiliations:** Department of Biological Psychology, Netherlands Twin Registry, Vrije Universiteit, Van der Boechorststraat 7, 1081BT Amsterdam, The Netherlands

**Keywords:** Exercise behavior, Heritability, Household, Spousal resemblance, Pedigree, Twins

## Abstract

We investigated the familial clustering of different classes of voluntary regular exercise behavior in extended twin-family pedigrees. In contrast to the earlier work based on twin data only, this allowed us to estimate the contributions of shared household effects (C), additive (A), and non-additive (D) genetic effects on voluntary exercise behavior. To test whether shared household effects were inflated by assortative mating we examined the causes of spousal resemblance. For adolescent and adult participants (aged 16 to 65) in the Netherlands Twin Register we constructed 19,543 pedigrees which specified all relations among nuclear family members and larger families in the register (N = 50,690 individuals). Data were available on total weekly MET minutes spent on leisure time exercise, and on total weekly MET minutes spent on exercise activities in team-based, solitary, competitive, non-competitive, externally paced and internally paced exercise. We analyzed the data in the Mendel software package (Lange et al. in Bioinformatics 29(12):1568–1570, 2013) under multiple definitions of household sharing and used data from spouses of twins to test phenotypic assortment, social homogamy, and marital interaction as potential sources of spousal resemblance. Results confirmed the influence of genetic factors on the total volume of weekly exercise behavior throughout the life span. Broad sense heritability ranged from 34 to 41% (19–26% A, 12–21% D), and did not depend on the definition for household sharing. Engaging in team-based, competitive, externally paced activities (e.g., soccer) was ~ 13% more heritable than engaging in non-competitive, solitary activities (e.g., jogging). Having shared a household as siblings explained 4–8% of the variance in adult exercise behavior, whereas sharing a household by spouses yielded higher C estimates (20–24%), as it incorporates spousal resemblance. Spousal resemblance was explained by both social homogamy and marital interaction, with little evidence for phenotypic assortment. We conclude that both the amount of voluntary exercise behavior and the preference for specific classes of exercise activities in adults is explained by additive and non-additive genetic factors and unique environmental influences that include correlated exercise behavior of spouses.

**Electronic supplementary material:**

The online version of this article (10.1007/s10519-019-09990-7) contains supplementary material, which is available to authorized users.

## Introduction

Regular voluntary exercise activities in leisure time, such as jogging, swimming, exercising at fitness clubs, and participation in recreational or competitive team and individual sports, are rapidly becoming the major source of physical activity of moderate-to-vigorous intensity in many industrialized countries. Recent meta-analyses of twin studies revealed broad sense heritability estimates for voluntary exercise behavior in adolescence and adulthood of 48% for males and 51% for females (van der Zee and de Geus 2019), with inconsistent evidence for an effect of shared environmental factors.

Most of the studies included in these meta-analyses had shortcomings that need to be addressed to increase our understanding of the causes of individual differences in this crucial health behavior. First, the vast majority of the studies base their conclusions on the classical twin model comparing monozygotic (MZ) and dizygotic (DZ) twin correlations. While producing robust estimates for broad sense heritability, this model cannot simultaneously estimate all four variance components potentially influencing exercise behavior: additive (A) and non-additive (D) genetic factors and shared (C) and unique (E) environmental factors. Choosing to set either non-additive or shared environmental variance components to zero can yield biased estimates, “sometimes wildly so” (Keller et al. 2009, p. 377). Second, previous studies have combined all recorded voluntary exercise activities into a single summary score, lumping together rather different activities like football, jogging, hockey, weight, strength training in fitness centers, tennis, swimming, boxing etc. Preference for these activities may, however, depend on rather different dispositional traits, both in terms of personality and in the physical, cognitive and motor skill demands placed on the individual.

In the current paper we assessed the sources of variation in voluntary regular exercise behavior using an extended twin-pedigree study design using a rich dataset from the Netherlands Twin Register (NTR). The NTR repository contains exercise data collected with virtually the same methods in twins, their parents, siblings, spouses and the children of twins and siblings. This dataset is therefore ideally suited for an extended twin family design. Such a design allows for the simultaneously estimation of additive and non-additive genetic factors and shared and unique environmental factors (Keller et al. 2009). The often used analytical approach for this estimation is structural equation modeling (SEM) software such as Mx, OpenMx, Mplus, or LISREL (Jöreskog 1970; Muthén et al. 2006; Neale et al. 1994). These programs offer great flexibility in model specification but are less well suited for large and irregular pedigrees where the specification of the complex multidimensional parameter space, as well as iteratively fitting the model to actual data, becomes quite challenging (Boomsma et al. 2018).

We previously used the Mendel software package (Lange et al. 2013) as an alternative approach to the analysis of data from large and complex twin-pedigrees (Boomsma et al. 2018). Mendel constructs the genetic relations among all pedigree members, e.g., MZ twin, sibling, cousins, aunt–niece, grandparent–grandchild, etc., and estimates components of genetic covariance for any pair of relatives from the weighted combination of additive and dominance effects (Weiss 1993). Using Mendel yielded estimates of additive genetic and dominance variance components for neuroticism of 24% and 19%, respectively (Boomsma et al. 2018). These results are consistent with those produced by SEM modeling in extended twin kinships among 45,850 family members from Australia and the United States (Lake et al. 2000).

In an extended twin-pedigree study design, the presence of spouses of MZ and DZ twins further allows us to address the sources of spousal resemblance (Eaves 1979; Heath and Eaves 1985; Van Grootheest et al. 2008). Spousal resemblance has been reported for voluntary exercise behavior with a median spouse correlation of 0.28, varying between 0.05 and 0.49 depending on the exercise trait considered (de Moor et al. 2011; Horimoto et al. 2011; Koopmans et al. 1994; Maia et al. 2014; Pérusse et al. 1988, 1989; Seabra et al. 2008, 2014). Spousal resemblance can be caused by the preferential mating of regular exercisers with other regular exercisers. This phenotypic assortment is based on a direct preference for similar traits and behaviors in a potential mate. Two well-known alternative mechanisms can cause spousal resemblance with respect to exercise behaviors. Social homogamy refers to mate selection based on sharing the same social milieu. Exercise behaviors scale positively with socioeconomic status, which in turn scales with neighborhood safety, higher mixed land use, and more exercise facilities. All of these may increase exercise behavior, although the effect sizes are rather modest (D'Haese et al. 2014; Gidlow et al. 2006). A third cause of spousal resemblance in exercise behavior is marital interactions. In this case, the longer spouses influence each other, the more similar they become. Note this can go both ways. Non-exercising partners may cause exercisers to swap out exercise activities for other social activities. Alternatively, the regular exercise activities of the exercising partner may become contagious for the non-exercisers. A telltale sign of marital interaction is that longer relationships are associated with greater similarity in exercise behaviors of the spouses.

The causes of spousal resemblance have consequences for the interpretation of genetic and unique and shared environmental variance estimates in an extended twin design. If spousal resemblance is caused by the preferential mating of exercisers with other exercisers it will increase parent–offspring and (non MZ twin) sibling similarity. In the classical twin design, this will cause inflated estimates for shared environmental effects (Falconer and Mackay 1996). Under social homogamy, the genetic resemblance between parents and offspring or between siblings is not expected to increase (Eaves et al. 1989). Marital interaction does not have consequences for genetic similarity in the next generation with the caveat that the increase in exercise similarity with marriage duration can be correlated with resemblance at mating: those who are more similar to each other at the start of the relationship may be more likely to remain together (Caspi and Herbener 1993).

**Fig. 1:**
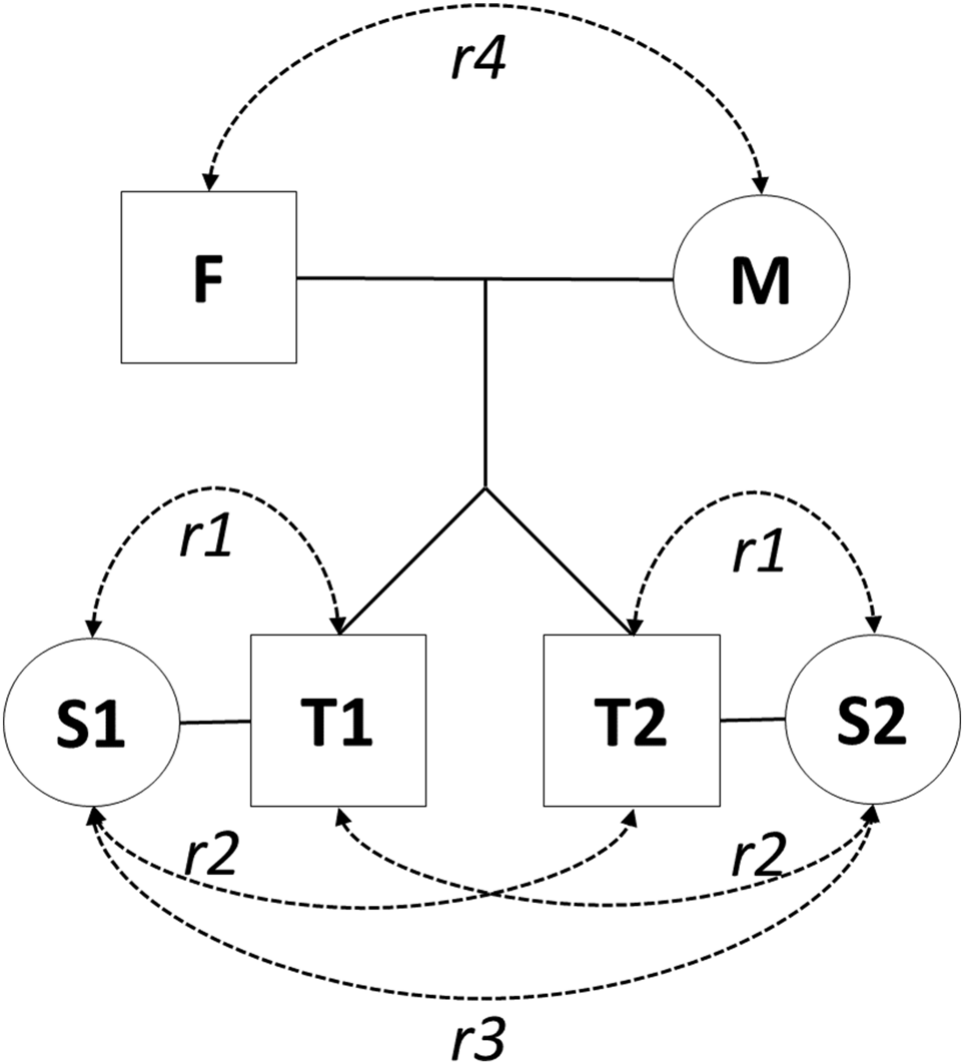
Model to analyze sources of spousal resemblance (Van Grootheest et al. 2008)

To test the presence and the causes of spousal resemblance, Van Grootheest et al. (2008) employed a design first coined by Reynolds et al. (2006) that is illustrated in Fig. 1. Given data in MZ and DZ twin pairs and their spouses, this method allows one to test for the presence of phenotypic assortment, social homogamy, and marital interaction by comparing the twin-spouse correlations (*r*_*1*_), co-twin spouse-correlations(*r*_*2*_), spouse1-spouse2 correlations (*r*_*3*_), and parent-parent correlations (*r*_*4*_) in pedigrees with data for twins and their spouses. If phenotypic assortment drives spousal resemblance then the expected pattern of correlations is *r*_*1*_ > *r*_*2*_ > *r*_*3*_, and—provided the trait is heritable—the resemblance between co-twin and spouse (*r*_*2*_) and between the twins’ spouses (*r*_*3*_) should be larger in MZ twins than in DZ twins. If, on the other hand, these correlations are equal (*r*_*1*_ = *r*_*2*_ = *r*_*3*_) or conform to (*r*_*1*_ = *r*_*2*_ > *r*_*3*_) and the comparison of *r*_*2*_with *r*_*3*_ does not differ for MZ and DZ families this suggests non-random assortment driven by social homogamy (Reynolds et al. 2000). Marital interaction would show as a larger resemblance in the older parent-of-twins couples compared to the younger twins-spouse couples, i.e. *r*_*4*_ should be greater than *r*_*1*_, assuming no other generational effects.

In the NTR, participants list the exact type of individual sports and exercise activities they engage in, in addition to the weekly frequency and duration. Performing the analyses per exercise activity, while arguably desirable, comes with the disadvantage of reducing the number of informative pedigrees. This holds even for the activities that are most frequently endorsed (jogging, fitness, soccer, tennis, swimming). However, exercise activities can be classified in six general domains using the three axes of solitary vs team-based activities, competitive vs non-competitive activities, and activities that are internally paced vs those that are externally paced (van der Mee et al. 2017). In competitive exercise, only activities with a competitive nature are included, i.e. a type of sport where a team or individual competes against another team or individual to win (e.g. soccer, tennis), whereas in non-competitive exercise only sports without a direct competitive nature are included (e.g., hiking, fitness). Team-based exercise reflects the volume of exercise activities spent in team-based sports, where multiple individuals work together to achieve a shared objective (e.g. field hockey, aerobic dance classes). Solitary exercise includes only the exercise or sports activities performed individually (e.g. jogging, swimming). Externally paced exercise represents the exercise behavior in activities in which the pace is determined by external factors, such as other participants or the weather conditions (e.g. judo, basketball, wind surfing). Internally paced exercise represents the volume of exercise behavior in activities in which the pace is determined by the exercisers themselves (e.g. tour cycling, inline skating, Nordic walking).

Here, we analyzed the total weekly volume of exercise (to be able to compare our results to previous findings), but also the volumes of weekly exercise behaviors specific to the six classes of exercise activity (competitive, non-competitive, team-based, solitary, externally paced, and internally paced). Based on previous twin and family studies, we expected to find a substantial broad sense heritability for the total volume of voluntary exercise behaviors, which should be mainly additive in nature. We further expected heritability of team-based, competitive and externally paced activities (field hockey, soccer) to be higher than that of solitary, non-competitive and internally paced activities (running, swimming). Enjoyment of team-based, competitive and externally paced activities (field hockey, soccer) is associated with physical fitness characteristics and cognitive and motor skills. Voluntary engagement in these activities therefore should reflect the heritability of these traits, which is known to be high (Schutte et al. 2016a, b). Finally, based on previous findings using a dichotomous trait for exercise participation in a subsample of the current data (de Moor et al. 2011), we expected phenotypic assortment to play a main role in spousal resemblance.

## Methods

### Participants

The NTR is an ongoing national initiative focused on data-collection in twins and their extended family members. Detailed information on the longitudinal data collection procedures in the NTR can be found elsewhere (Ligthart et al. 2019). Every two to three years, extensive surveys are sent to parents of young twins, adult and adolescent twins and their parents and spouses. Almost all of these surveys included comparable questions on the type and volume of their exercise behaviors. For the present analyses, we used surveys filled out by participants in the age range of 16 to 65 years. Participants may have filled out surveys in multiple waves. For our present purposes, we selected one, if several data at waves were available. Specifically, we selected the wave in which the largest number of family members filled out the survey. For family members who did not participate in the chosen wave, we used the survey closest in time. If the family members differed greatly with respect to survey, we selected the last survey each member filled out. Finally, we only included participants who had at least one other participating family member (in any wave). Spouses of twins here count as family members. Throughout, we use ‘spouse’ to indicate the “partner with whom you share a lasting and stable relationship (like a marriage)”, which was the wording used in the surveys.

The number of nuclear families with exercise data and with at least two family members

was 19,543, and the total number of participants with valid exercise data was 50,690. Table 1 describes the composition of these NTR pedigrees and their age characteristics. A more complex pedigree structure in some families was available than these nuclear family relationships. For instance, there was information on 18 grandparents of twins. There were seven large (≥ 20 family members) pedigrees. The Mendel software fully exploits these complex family structures, providing extra sources of information, for the estimation of A, D, and C beyond that provided by parents, twins and siblings.

**Table 1:** Composition of families

	N	Mage	SDage
Families^a^	19,543	–	–
Individuals	50,690	38.63	13.65
MZ males	2560	27.26	12.41
MZ females	5201	29.88	13.63
DZ males from DZ male pairs	1680	24.86	11.14
DZ females from DZ female pairs	2976	27.20	12.00
DZ individuals from opposite-sex pairs	4293	25.03	10.40
Fathers	12,674	48.21	7.41
Mothers	15,653	45.89	7.70
Brothers	5268	26.72	11.75
Sisters	7982	28.34	12.51
Spouse-pairs with offspring^b^	13,881	46.80	7.54
Spouse-pairs without offspring^b^	1976	36.04	14.54

N: generally denotes number of individuals unless indicated by superscript

^a^N = number of pedigrees with at least 2 individuals

^b^N = number of spouse-pairs

### Voluntary exercise behavior

Voluntary exercise behavior was assessed using survey questions in nearly all NTR questionnaires discussed above. Participants were first asked whether they regularly participate in exercise in their leisure time (“Yes” or “No”). If the response was affirmative they were asked (1) which sport they participate in, (2) how many years they have been doing so, (3) how many months a year they do so, (4) how many times a week on average they do so, (5) how many minutes on each occasion they exercise on average. Metabolic equivalent of task (MET) values were assigned to each exercise activity following Ainsworth et al. (2011). This MET value reflects the energy expenditure of the type of exercise as a multiple of basal energy expenditure (approximately 1 kcal/kg/h). Using these data, all exercise activities were assigned a weekly MET-minutes value by calculating the product of (1) the intensity as the MET value, (2) the weekly frequency, and (3) the average duration in minutes. The total volume of exercise behavior of the participant (METmin) was the sum of the weekly METminutes across all exercise activities. Obligatory exercise such as physical education (PE) classes, or other non-voluntary activity such as biking as a form of transportation, as well as seasonal exercise, such as skiing during winter only, were excluded, as they are not part of regular voluntary exercise behavior. Weekly METmin scores for the exercise domains were calculated in a similar fashion, but with selective inclusion of the types of exercise activities that fit within each of the domains, i.e. competitive or non-competitive team-based or solitary activities, and internally or externally paced activities. The complete list of classification for each type of exercise is detailed in Supplementary Table I. Test–retest reliability of total METmin was 0.82 as computed among 200 individuals who completed the questions about exercise in December 2004 and again 6 months later. Furthermore, using analysis of tracking coefficients of total METmin as well as the scores across the six domains showed moderate to high temporal stability for total volume of exercise across periods varying from 2 to 22 years, ranging from 0.38 to 0.77 with a median of 0.57 (van der Zee et al. 2019a, b).

### Statistical analyses

To obtain a first impression of familial resemblance for exercise behaviors, correlations were estimated for MZ and DZ twin pairs, for biological sibling pairs, for father–offspring and for mother–offspring pairs, and calculating the Pearson’s correlation of the METmin variables of the two. This was repeated for every exercise domain. Since we have previously found significant sex, age and sex*age effects on the total volume of exercise behavior (van der Zee et al. 2019a, b), we first used linear regression to correct for age, sex and the age*sex effects on exercise behavior. The Pearson’s correlations were based on the regression residuals. Two different spouse correlations were computed: one for parents of twins and one for twins and their spouses, who on average were 9 years younger than the parents of twins.

Variance components attributable to additive genetic factors (A), household influences (C), non-additive genetic factors (D), and unique environmental factors (E) were estimated using the Mendel software package (Lange et al. 2013). In all analyses, age, sex and age*sex were included as fixed effects rather than regressing them out beforehand as we did for the kinship correlations. As detailed in Boomsma et al. (2018), the algorithm, which takes the NTR administrative database as input, provides as output the pedigree structures in standard pedigree format, as used in software, such as Mendel or Merlin (Abecasis et al. 2002). Mendel has an obligatory field to indicate the presence of MZ twins in the pedigree. Optionally, extra fields can be included, to indicate whether persons share the same household within a pedigree. Whereas genetic relations do not change, household sharing can depend on the time of assessment of the exercise behaviors. Dutch offspring typically share a household with their parents and siblings (if any) until around age 18. After about 18 they establish their own households, which can be shared with their own spouse. Sharing a household was defined in four different ways:

- Full Household: These household factors contribute to the resemblance of spouses, all parent–offspring pairs, and all twin and non-twin siblings pairs, with twin and non-twin offspring restricted to those under 18. Note that these effects are treated as age and birth-cohort invariant, i.e., they are the same for twins/siblings and their spouses in the pedigree as for the parents of twins/siblings.
- Spouse Household: These household factors only contribute to spouse resemblance.
- Sib Household: These household factors only contribute to the resemblance of twins and non-twin siblings that have shared a household up to age 18.
- Twin Household: These household factors are specific to twins who have shared a house hold up to age 18.

To test for the sources of spouse resemblance, we used the approach suggested by Van Grootheest et al. (2008), as shown in Fig. 1, and briefly outlined in the introduction. Full maximum likelihood estimation was used in OpenMx (Neale et al. 2016) to obtain the variance–covariance matrix for the six family members expressed in Fig. 1, separately for MZ, same-sex DZ twin families. Opposite-sex DZ twins were excluded from this analysis as the opposing sex of twins and their spouses would bias the results of *r*_2_ and *r*_*3*_, whereas in MZ and same-sex DZ twins the comparison is always either between same-sex or different-sex pairs. Age, sex, and the age*sex were included as covariates in this model, and all correlations were constrained to be equal across MZ and DZ twins when no direct comparison was made (i.e. *r*_*1*_, *r*_*4*_, the four parent offspring correlation, and a residual correlation of parents to offspring-spouses). The correlations of interest obtained from the estimated variance–covariance matrix convey the resemblance between twins and their spouses (*r*_*1*_), twins and the spouses of their co-twins (*r*_*2*_), the spouse of twin 1 and the spouse of twin 2 (*r*_*3*_), and the parents of the twins (*r*_*4*_). First, to confirm the presence of spousal resemblance, both the hypotheses *r*_*1*_= 0 and *r*_*4*_=0 were tested by means of a 2-df likelihood ratio test. Second, to test for phenotypic assortment and social homogamy, four hypotheses were tested by means of 1 df likelihood ratio tests: (1) *r*_*1*_is equal to *r*_*2*_, vs. *r*_*1*_ > *r*_*2*_; (2) r_2_ is equal to *r*_*3*_, vs. *r*_*2*_ > *r*_*3*_; (3) *r*_*2*_in MZ twins equals *r*_*2*_in DZ twins, vs. *r*_*2*_ in MZs > *r*_*2*_ in DZs; and (4) *r*_*3*_in MZ twins equals *r*_*3*_in DZ twins, vs. *r*_*3*_ in MZs > *r*_*3*_ in DZs. Significant effects (*p* < 0.05) favoring these four hypotheses were considered evidence for phenotypic assortment. Rejection of these hypotheses is considered to be evidence for social homogamy. The presence of marital interaction was tested by a 1 df likelihood ratio test of *r*_*4*_ = *r*_*1*_, vs. *r*_*4*_ > *r*_*1*_.

As a post-hoc test of the influence of phenotypic assortment on the estimates for the narrow-sense heritability (i.e. the additive genetic variance component) we performed mid-parent to offspring regression. We computed the mean parental values for exercise behavior (correct for age, sex and age*sex), and regressed these on the same exercise phenotype in one randomly selected child per family. In contrast to other estimations of narrow-sense heritability, this test is not sensitive to phenotypic assortment, and will not lead to biased estimation of additive genetic variance (Falconer and Mackay 1996). Because the estimates for A for exercise behavior have been shown to decrease across the adult life span (Vink et al. 2011), we computed mid-parent to offspring regression in four age bins (20–45, 46–50, 51–55, 55–70 years).

## Results

Means and standard deviations of the various exercise domains, as well as age for each family relation (parent, child, or twin) split by sex are included in Supplementary Table II. All kinship correlations and the number of pairs in each combination are presented in Table 2. For total METhr, monozygotic (MZ) twin correlations were 0.58, and same-sex dizygotic (DZ) twin and sibling correlations varied between 0.31 and 0.42, with little evidence of sex differences. Opposite sex DZ twin and brother-sister sibling correlations were slightly lower (~ 0.22). Parent–offspring correlations were between 0.12 and 0.23, which might reflect that parents and offspring share 50% of the additive genetic variance, but in contrast to DZ and sibling pairs no genetic dominance variance. The same pattern of results characterizes the other exercise domains with generally stronger familial resemblance estimates for team-based, competitive, and externally paced activities than for solitary, non-competitive, and internally paced activities.

**Table 2:** Kinship correlations

Correlation	N_pairs_	Total	Team	Comp	Sol	Ncomp	PaceI	PaceE
All Spouses	13,002	0.31	0.50	0.42	0.31	0.30	0.32	0.44
Parents of twins	11,879	0.32	0.52	0.45	0.32	0.30	0.32	0.46
Twins—spouses^a^	1767	0.28	0.37	0.31	0.29	0.26	0.29	0.33
MZM	1280	0.58	0.70	0.67	0.50	0.52	0.42	0.67
MZF	2589	0.58	0.77	0.72	0.56	0.55	0.43	0.72
DZM	824	0.37	0.55	0.45	0.44	0.52	0.32	0.45
DZF	1468	0.42	0.58	0.53	0.42	0.42	0.28	0.54
DOS	1857	0.23	0.35	0.24	0.23	0.23	0.20	0.27
Brother–brother	862	0.31	0.51	0.40	0.38	0.46	0.28	0.40
Brother–sister	3281	0.22	0.30	0.23	0.19	0.19	0.16	0.24
Sister–sister	1885	0.39	0.58	0.51	0.35	0.35	0.25	0.52
Mother–daughter	11,451	0.23	0.38	0.32	0.22	0.22	0.20	0.33
Mother–son	11,025	0.12	0.16	0.12	0.18	0.20	0.14	0.13
Father–daughter	9465	0.21	0.34	0.27	0.20	0.20	0.17	0.29
Father–son	9137	0.20	0.20	0.19	0.22	0.23	0.20	0.19

*MZM* monozygotic male pairs, *MZF* monozygotic female pairs, *DZM* dizygotic male pairs, *DZF* dizygotic female pairs, *DOS* dizygotic opposite sex pairs, *Total* Total METmin, *Team* Team METmin, *Comp* Competitive METmin, *Sol* Solitary METmin, *Ncomp* Non-competitive METmin, *PaceI* internally paced METmin, *PaceE* Externally paced METmin

^a^If twins had twin offspring they were used in this row only, and not reused as parents of twins

### Mendel’s estimates of genetic and environmental effects

**Fig. 2:**
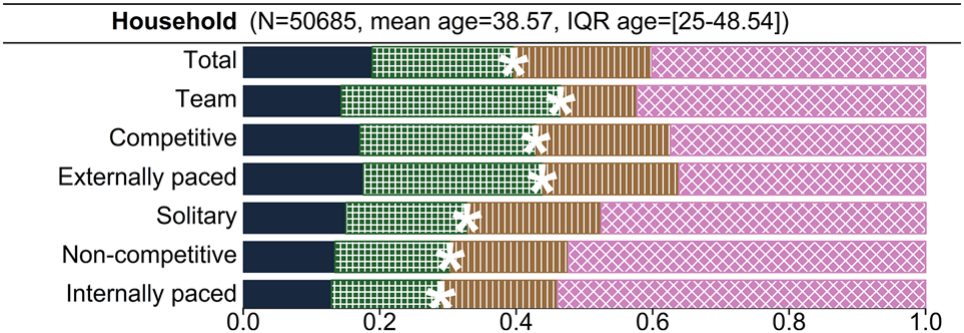
Proportion of variance explained by additive genetic (A), non-additive genetic (D), shared environmental (C), unique environmental (E) factors and broad sense heritability (*) for different classes of exercise. The C component reflects the environment shared by spouses

Full results for all exercise domains and models tested are included in Supplementary Table III. In all exercise domains, the best fitting model was the model including additive genetic, non-additive genetic (dominance), household, and unique environmental variance (ACDE) components. This was true across all four household definitions, indicating that heritability estimates are not very sensitive to how a shared household component is modelled. A summary of the model using the full household definition is presented in Fig. 2, and a representation of the other models is presented in Supplementary Fig. 1. Broad-sense heritability of total METmin was 40%. The broad-sense heritabilities of team-, competitive- and externally paced METmin (46%, 43%, and 44%, respectively) were consistently higher than the broad-sense heritabilities of solitary-, non-competitive-, and internally paced METmin (33%, 30%, and 29%, respectively). These increases in heritability are largely explained by an increase in non-additive genetic effects. The effects of shared household components largely depended on its definition, with estimates of C for, e.g. total volume being low in the sibling (4%) and twin (8%) household models, and moderate in the full (20%) and spousal (24%) household models. The C component in the latter two models was driven by the spousal correlations.

### Causes of spousal resemblance

The ML estimate of twin—spouse correlation for total METmin was 0.19 in the subsample used for the analysis of sources of spousal resemblance. The average age of the twins and spouses in this subsample was 37.3 years. Spouse correlations in parents of twins, who were on average 46.8 years old, were significantly higher (r = 0.35, *p* = 5.77 × 10^−12^), indicating marital interaction may contribute to spousal resemblance. Table 3 contains a summary of the hypotheses tested for the cause of spousal resemblance in the total volume of exercise behavior. Similar tables for the six different types of exercise activities can be found in Supplementary Tables IV–IX. Spousal resemblance was significant across all exercise phenotypes (*p’s* < 0.05) which is in line with the results from the different household models assessed in Mendel. The hypothesis of marital interaction, i.e., the correlation between spouses living together for a longer time (parents of twins) is larger than the correlation between those living together for a shorter time (twins and their spouses), was also supported across *all* exercise domains. The effect of marital interaction appeared to be particularly strong for team-, competitive- and externally paced sports activities.

**Table 3:** Spousal resemblance and its sources in total volume of exercise

Hypothesis	N	Estimates	Δ-2LL	Δ*df*	*p*	Spousal Resemblance	Phenotypic Assortment	Social Homogamy	Marital interaction
*r1* > 0	1608	0.20 > 0	82.46	1	1.08 × 10^−19^	√			
*r4* > 0	10,711	0.35 > 0	825.05	1	1.94 × 10^−181^	√			
*r1* > *r2*	1420	0.20 > 0.11	17.02	2	2.02 × 10^−4^		√	–	
*r2* > *r3*	419	0.11 > 0.08	0.69	2	0.71		–	√	
*r2*_*MZ*_ > *r2*_*DZ*_	465	0.11 > 0.11	0.01	1	0.92		–	√	
*r3*_*MZ*_ > *r3*_*DZ*_	157	0.06 > 0.12	0.39	1	0.53		–	√	
*r4* > *r1*	1608	0.35 > 0.20	47.41	1	5.77 × 10^−12^				√

Hypothesis: expectations for the patterns of twin-spouse correlations (*r*_*1*_), co-twin spouse-correlations(*r*_*2*_), spouse1-spouse2 correlations (*r*_*3*_), and parent-parent correlations (*r*_*4*_) under phenotypic assortment, social homogamy, and marital interaction. Under phenotypic assortment the expected pattern of correlations is *r*_*1*_ > *r*_*2*_ > *r*_*3*_, *r2*_*MZ*_ > *r2*_*DZ,*_* r3*_*MZ*_ > *r3*_*DZ*_. Under social homogamy the expected pattern is (*r*_*1*_ = *r*_*2*_ ≥ *r*_*3*_), *r2*_*MZ*_ = *r2*_*DZ,*_* r3*_*MZ*_ = *r3*_*DZ*_. Under marital interaction we expect *r4* > *r1;* N: Number of complete pairs in the data (if two correlation coefficients are compared the lowest N is presented); Δ-2LL: Difference in -2 log-likelihood compared to the base model; Δdf: Difference in degrees of freedom compared to the base model, and used in the chi-squared test; *p*: p-value of the chi-squared difference test comparing the base model to the constrained model; √: Hypothesis supported with α = 0.01

Across all exercise domains, the support for social homogamy was greater than the support for phenotypic assortment. Evidence for phenotypic assortment was limited to the greater resemblance of twins and their own spouses (*r*_*1*_) than the resemblance of twins and the spouses of their co-twin (*r*_*2*_). We note that our data cannot discriminate between assortment at the time of mate selection and marital interaction as the source of this higher resemblance.

### Influence of phenotypic assortment on additive genetic effects

Regression coefficients obtained in the mid-parent offspring regression on all exercise phenotypes are displayed in Table 4. These provide a direct estimate of the narrow-sense heritability and are presented along with the narrow-sense heritability estimates obtained in the full household model analysis in Mendel. The regression coefficients from mid-parent regression in each phenotype were very close to the narrow-sense h^2^ estimates from the extended family model, at each age bin tested. This shows that if phenotypic assortment was present, it had little to no influence on the Mendel estimate of the A variance component.

**Table 4:** Narrow sense heritability, estimated based on mid-parent offspring regression and from modeling full pedigree data in Mendel

Parental age cutoffs	20–45 years	45–50 years	50–55 years	55–70 years	Mendel narrow-sense h^2^
M_age_ parents	42.67	47.85	52.59	59.68	
M_age_ offspring	19.00	20.09	22.08	27.48	
N_pairs_	725	1369	1463	1238	
Total exercise	0.22	0.18	0.22	0.18	0.19
Team-based	0.21	0.18	0.13	0.14	0.14
Competitive	0.27	0.21	0.19	0.12	0.17
Externally paced	0.23	0.19	0.18	0.13	0.18
Solitary	0.23	0.15	0.19	0.19	0.15
Non-competitive	0.22	0.13	0.18	0.13	0.13
Internally paced	0.21	0.13	0.18	0.10	0.13

N_pairs_: Total number of complete pairs (at least one parent and at least one child) used in the regression. Age bins are based on the mean age of the parents. Estimates represent the coefficient from linear regression. Estimated A represents the estimate of A from the extended-family model tested in Mendel

## Discussion

We examined the sources of individual differences in voluntary exercise behaviors in the Netherlands using an extended twin pedigree design. Broad sense heritability for total volume of exercise estimates ranged from 34 to 41% in models using four different definitions of household sharing. Across the various classes of exercise activities, genetic contribution was largest to team-based, competitive and externally paced activities (39% < h^2^ < 47%) with lower broad sense heritability for solitary, non-competitive and internally paced activities (26% < h^2^ < 34%). These differences in heritability are largely explained by a larger component of non-additive genetic effects for team-based over solitary activities, a 7% increase for competitive over non-competitive activities, and a 8% increase for externally over internally paced activities. Results from the extended twin pedigree design further suggested that a shared sibling household (typically up till age ~ 18) explains 4–8% of the variance in adult exercise behavior, whereas sharing a household by spouses yielded higher estimates of the C variance component (20–24%) as it had to incorporate the spousal resemblance. The total spouse correlation was 0.32 for total exercise behavior and varied between 0.30 and 0.50 across the specific classes of exercise activities, which was best explained by marital interaction and social homogamy, with weaker evidence for phenotypic assortment.

Our broad sense heritability estimates for voluntary exercise behavior (40%) is 10% lower than the average of 50% obtained in twin registries from multiple countries (van der Zee and de Geus 2019), which we believe reflects the larger numbers of adolescents and young adults in these studies. Heritability of voluntary behavior has been shown to peak in late adolescence (~ 65%) (Huppertz et al. 2016) and then gradually decrease across the life span (Vink et al. 2011).

A new finding in the current study is the clear evidence for the contribution of non-additive effects to the broad sense heritability, most prominent for team-based, competitive and externally paced activities. Previous use of extended twin designs for a variety of traits has similarly found higher non-additive and lower additive effects for behavioral traits than the classical twin design (Coventry and Keller 2005). These non-additive genetic effects provide an explanation for the large discrepancy that has been found in the heritability estimates deriving from parent-offspring/sibling correlations in family studies compared to MZ/DZ twin based estimates in twin studies. Family studies (de Moor et al. 2011; Horimoto et al. 2011; Koopmans et al. 1994; Maia et al. 2014; Pérusse et al. 1988, 1989; Seabra et al. 2008, 2014) have systematically reported 11% lower heritability estimates than twin-only studies. Such differences may be partly attributed to the difference in these estimates to non-additive genetic effects, which contribute to resemblances of monozygotic twins and full siblings but not to resemblance of parents and offspring or other relatives such as cousins. The contribution of non-additive genetic effects may also have implications for genome-wide association studies (GWAS), as the traditional GWAS assumes additive genetic effects rather than take non-additivity into account.

Previous studies in adults twins have reported only small shared environmental influences on voluntary exercise behaviors with a meta-analysis estimating them to be about 10% (van der Zee and de Geus 2019). As most of the studies in this meta-analysis used the classical twin design, the C estimates will largely reflect the sharing of environmental factors by the twins when they were still living at home. In our analyses, shared environmental effects were modeled in four different ways depending on the timing of survey completion: sharing a household as spouses at the time of survey completion, ever sharing a household either as siblings or as parents and offspring, sharing a household as siblings, or sharing a household as twins. The latter two definitions of shared environmental effects assume that such effects linger after twins and siblings are separated from each other and form their own households (unshared by relatives). Our results using the SibHousehold and TwinHousehold models support the existence of a small impact of a sharing a household until late adolescence on the total volume of exercise behavior, with estimates (4–8%) corresponding quite well with the meta-analysis.

In contrast, much larger shared household effects were found (20–24%) when using the SpouseHousehold or FullHousehold models. We surmise that these shared household effects derive largely from the spouse correlations in the twin/sibling and parental generations, which mostly reflected marital interaction across all types of exercise behaviors, including total exercise behavior. The latter contrasts with previous studies that did not find marital interaction for yes/no exercise participation (de Moor et al. 2011). The exercise-activity specific marital interaction suggests that the exercise behavior of a spouse constitutes an important environmental factor for the exercise behavior of a person. We note that marital interaction was weaker for solitary, non-competitive, internally paced activities than for team-based, competitive and externally paced activities. We further note that people tend to engage much less in team-based and competitive exercise activities over time and that solitary, non-competitive, internally paced activities are the largest source of adult exercise participation (van der Zee et al. 2019a, b). It may simply be difficult to maintain the commitments required for team-based and competitive activities without symmetrical interest and engagement of one’s partner.

Marital interaction processes give rise to genotype-environment covariance, which can produce apparent shared environmental influences (Dolan et al. 2014) at the expense of additive genetic influences. Likewise, if not explicitly modeled, phenotypic assortment may also affect A estimates, although spousal resemblance has to be fairly large to achieve noticeable bias. Phenotypic assortment can be discriminated from social homogamy by comparing the resemblances in twin and co-twin spouses (*r*_*2*_) and in twin spouses and co-twin spouses (*r*_*3*_) as a function of zygosity. Phenotypic assortment will make the spouses of MZ twins more alike to their co-twin and to each other than is found in the spouses of DZ twins. If non-random assortment is driven by social homogamy, *r*_*2*_ and *r*_*3*_ will *not* differ between MZ and DZ twins. Across all exercise phenotypes, social homogamy was favored over assortment at mate selection as the main source of spousal resemblance, but we note that social homogamy is tested as the null-hypothesis and power for detecting small-sized phenotypic assortment was low. Converging evidence that phenotypic assortment plays a minor role came from the mid-parent offspring regression analyses. The mid-parent offspring regression yields an estimate of narrow-sense heritability that is insensitive to assortment (Falconer and Mackay 1996). Comparison of these estimates to the heritability estimates from Mendel showed that the latter were not overly biased by phenotypic assortment.

We acknowledge various limitations in our approaches. First, our results are based on twin families, and we do not know if and to what extent determination of exercise behavior in these families differs from that in non-twin families. However, we have noted previously (van der Zee et al. 2019a, b) that there are no differences between the twins and the singleton siblings, and that the prevalence of the MET scores for regular voluntary exercise behavior in the Dutch twin families is very consistent with the prevalence of voluntary leisure time physical activity reported by 5 large-scale studies in Europe. Thus the results of the current study may be generalizable to the Dutch population at large.

Second, the way we carved up the total weekly volume into the six classes of exercise activity (competitive, non-competitive, team-based, solitary, internally paced, and externally paced) is far from ideal. Although not completely overlapping, these classes were not independent, in that the same activity, e.g. soccer would count in the team-based, competitive, and externally paced (paced) weekly exercise volumes.

A third limitation is the assumption that the same genes are expressed across sex and age. Parents and offspring’s phenotypes were assessed at different ages. If the genetic correlation across the lifespan would be less than one for phenotypes that are analyzed this would result in a ‘too low’ parent–offspring correlation, compared to the sibling and DZ pair correlations, potentially causing a wrong conclusion about the presence of genetic dominance. There was some suggestion for a sex limitation model, but less so in the parent–offspring than in the sib data.

Fourth, we used the Mendel software as an efficient way to model the multigenerational familial resemblances, accepting that some effects could not be specified in the genetic model. A different approach would have been to use the ‘Cascade’ model (Keller et al. 2009). This model allows testing of all variance components as well as specific tests of social homogamy vs phenotypic assortment, and can even be made to incorporate vertical transmission (although we have encountered no actual examples of this). Using the simpler ‘Stealth’ model on the total volume of exercise behavior we previously found no evidence for vertical (or cultural) transmission suggesting that there is no direct ‘copying’ of parental exercise habits by the offspring (de Moor et al. 2011). This appears to be true for a large number of behavioral traits (Eaves 2009) and we assumed here that it also applies to the exercise activities in the specific classes. It could be reasonably argued that for team sports this assumption may not hold.

In summary, regular voluntary exercise behavior, regardless of what type of exercise, is a moderate to highly heritable trait with substantial contribution by non-additive genetic factors. Competitive- team-based and externally paced exercise activities appear to be more heritable compared to non-competitive, solitary and internally paced activities, largely due to an increased effect of these non-additive genetic factors. In childhood and (early) adolescence the environment shared by siblings is a major contributor to exercise behavior (Huppertz et al. 2016) but having shared that environment plays only a minor role in voluntary exercise behaviors in adulthood. In adulthood, the exercise behavior of one’s life partner becomes the more relevant environmental factor.

## Electronic supplementary material

Below is the link to the electronic supplementary material.

Supplementary file1 (DOCX 103 kb)

Supplementary file2 (TIFF 4243 kb)
