# Supplementary material for: An Extended Twin-Pedigree Study of Different Classes of Voluntary Exercise Behavior
Source: Behav Genet. 2020 Jan 23;50(2):94–104. doi: 10.1007/s10519-019-09990-7 (PMC7028831; doi:10.1007/s10519-019-09990-7)
Supplement: Supplementary file 1 — Supplementary file1 (DOCX 103 kb) [file 10519_2019_9990_MOESM1_ESM.docx]

**Supplementary Table I. Classification of various types of exercise**

| **Exercise domain** | **Subdomain** | **Reported activities** | **Excluded** | **Team sport** | **Competitive sport** | **Internal/External pacing** |
| --- | --- | --- | --- | --- | --- | --- |
| Aerobics | Aerobics | Aerobics |  | no | no | 4 |
|  |  | Combi-fit |  | no | no | 4 |
|  | High impact aerobics | Grit |  | no | no | 4 |
|  |  | High impact aerobics |  | no | no | 4 |
|  |  | HIIT |  | no | no | 4 |
|  |  | Insanity |  | no | no | 4 |
|  |  | Labooca |  | no | no | 4 |
|  |  | Tae Bo |  | no | no | 4 |
|  | Low impact aerobics | Aeronatics |  | no | no | 4 |
|  |  | BBB |  | no | no | 4 |
|  |  | Callanetics |  | no | no | 4 |
|  |  | Low impact aerobics |  | no | no | 4 |
|  | Steps | Body Steps |  | no | no | 4 |
|  |  | Steps |  | no | no | 4 |
| Acrobatics/gymnastics | Acrobatics | Acrobatic Rock-n-Roll |  | Can be | Can be | 3 |
|  |  | Acrobatics |  | Can be | Can be | 3 |
|  |  | Tumbling |  | Can be | Can be | 3 |
|  | Cheerleading | Cheerleading |  | yes | yes | 3 |
|  | Color Guard | Color Guard |  | Can be | Can be | 3 |
|  | Figure Scating | Figure Scating |  | Can be | YES | 3 |
|  | Gymnastics | Cesar therapy |  | no | no | 4 |
|  |  | Competitive gymnastics |  | Can be | yes | 4 |
|  |  | Gymnastics |  | no | no | 4 |
|  |  | Heart Disease Gymnastics |  | no | no | 4 |
|  |  | Mensendieck Gymnastics |  | no | no | 4 |
|  |  | Pregnancy gymnastics |  | no | no | 4 |
|  |  | Recovery exercises |  | no | no | 4 |
|  |  | Rhythmic gymnastics |  | Can be | no | 3 |
|  |  | Voltige |  | Can be | Can be | 3 |
|  | Platform diving | Platform diving |  | no | yes | 3 |
| Aiming based sports | Archery | Archery | yes | no | Can be | 4 |
|  | Billiards | Billiards | yes | no | yes | 4 |
|  | Bowling | Bowling | yes | Can be | yes | 4 |
|  |  | Jeu de boules | yes | Can be | yes | 4 |
|  |  | Petanque | yes | Can be | yes | 4 |
|  | Curling | Curling | yes | yes | yes | 4 |
|  | Darts | Darts | yes | no | yes | 4 |
|  | Frisbee | Frisbee |  | yes | Can be | 3 |
|  |  | Ultimate frisbee |  | yes | Can be | 3 |
|  | Golf | Golf |  | no | Can be | 4 |
|  | Shooting | Gun | yes | no | Can be | 4 |
|  |  | Rifle | yes | no | Can be | 4 |
| Air based sports |  | Hang gliding |  | no | no | 3 |
|  |  | Sky diving |  | no | no | 3 |
| Ball games | Baseball | Baseball |  | yes | yes | 1 |
|  |  | Softball |  | yes | yes | 1 |
|  | Basketball | Basketball |  | yes | yes | 1 |
|  |  | Basketball (wheelchair) |  | yes | yes | 1 |
|  |  | Basketball (supervised) |  | yes | yes | 1 |
|  | Cricket | Cricket |  | yes | yes | 1 |
|  | Handball | Handball |  | yes | yes | 1 |
|  | Hockey | Hockey |  | yes | yes | 1 |
|  |  | Hockey (wheelchair) |  | yes | yes | 1 |
|  |  | Ice Hockey |  | yes | yes | 1 |
|  |  | In-line Hockey |  | yes | yes | 1 |
|  |  | Water Hockey |  | yes | yes | 1 |
|  | Korfball play | Korfball play |  | yes | yes | 1 |
|  | Polo | Polo |  | yes | yes | 1 |
|  | Rugby | Rugby |  | yes | yes | 1 |
|  |  | Rugby (wheelchair) |  | yes | yes | 1 |
|  | Soccer | Beach Soccer |  | yes | yes | 1 |
| Ball games (cont.) |  | Floorball |  | yes | yes | 1 |
|  |  | Hall sports |  | yes | yes | 1 |
|  |  | Hall sports (supervised) |  | yes | yes | 1 |
|  |  | Indoor soccer |  | yes | yes | 1 |
|  |  | Indoor soccer (wheelchair) |  | yes | yes | 1 |
|  |  | Soccer |  | yes | yes | 1 |
|  | Volleyball | Beach Volleyball |  | yes | yes | 1 |
|  |  | Competitive volleyball |  | yes | yes | 1 |
|  |  | Volleyball |  | yes | yes | 1 |
|  |  | Volleyball (sitting) |  | yes | yes | 1 |
|  | Various | Ball games (other) |  | yes | yes | 1 |
| Cardiotraining/ | Athlons | Biathlon |  | no | yes | 3 |
| edndurance based |  | Pentathlon |  | no | yes | 3 |
|  |  | Triathlon |  | no | yes | 3 |
|  | Cardiotraining | Cardiotraining |  | no | no | 4 |
|  |  | Elliptical trainer |  | no | no | 4 |
|  |  | Rowing machine |  | no | no | 4 |
|  |  | Spinning |  | no | no | 4 |
|  |  | Water Spinning |  | no | no | 4 |
|  | Cycling | Cycling (race) |  | no | Can be | 4 |
|  |  | Handbiking |  | no | no | 4 |
|  |  | Mountainbiking |  | no | Can be | 4 |
|  | Endurance training | Competitive running |  | no | yes | 4 |
|  |  | Endurance training |  | no | no | 4 |
|  |  | Fast walking |  | no | Can be | 4 |
|  |  | Freerunning |  | no | Can be | 3 |
|  |  | Jogging |  | no | no | 4 |
|  |  | Jumping rope |  | no | no | 4 |
|  |  | Running |  | no | Can be | 4 |
|  |  | Treadmill |  | no | no | 4 |
|  |  | Trampoline jumping |  | no | no | 4 |
| Cardiotraining/ |  | Water jogging |  | no | no | 4 |
| edndurance based | Skating | In-line skating |  | no | Can be | 4 |
| (cont.) |  | Rollarskating |  | no | no | 4 |
|  |  | Skateboarding |  | no | Can be | 4 |
|  | Stationairy bicycling (90-100 watts) | Cycling (tours) |  | no | no | 4 |
|  |  | Hometrainer |  | no | no | 4 |
|  |  | Hypoxi |  | no | no | 4 |
| Climbing | Mountain Climbing | Mountain Climbing |  | no | no | 3 |
|  | Wall Climbing | Wall Climbing |  | no | no | 3 |
|  |  | Canyoning |  | no | no | 3 |
|  |  | Potholing |  | no | no | 3 |
| Dancing | Ballet (like) | Ballet |  | no | Can be | 3 |
|  |  | Jazz dancing |  | no | no | 3 |
|  |  | Modern dancing |  | no | Can be | 3 |
|  | Ballroom dancing | Ballroom dancing |  | no | Can be | 3 |
|  |  | Competitive ballroom dancing |  | no | yes | 3 |
|  |  | Latin dancing |  | no | Can be | 3 |
|  |  | Tango dancing |  | no | Can be | 3 |
|  | Cultural dancing | Belly dancing |  | no | no | 3 |
|  |  | Bio dancing |  | no | no | 3 |
|  |  | Body by Dance |  | no | no | 3 |
|  |  | Breakdance |  | no | no | 3 |
|  |  | Flamengo dancing |  | no | no | 3 |
|  |  | Hiphop dancing |  | no | no | 3 |
|  |  | Salsa dancing |  | no | no | 3 |
|  |  | Showdance |  | no | no | 3 |
|  |  | Streetdance |  | no | no | 3 |
|  | Dancing (general) | Country dancing |  | no | no | 3 |
|  |  | Dancing (unspecified) |  | no | no | 3 |
|  |  | Dancing (wheelchair) |  | no | no | 3 |
|  |  | Disco dancing |  | no | no | 3 |
| Dancing (cont.) |  | Folk dancing |  | no | no | 3 |
|  |  | Line dancing |  | no | no | 3 |
|  | Dancing aerobics | Aerobic dance |  | no | no | 3 |
|  |  | Rock & Roll dancing |  | no | no | 3 |
|  |  | Shabam |  | no | no | 3 |
|  |  | Zumba |  | no | no | 3 |
|  | Gogo dancing | Gogo dancing |  | no | no | 3 |
|  | Tapdancing | Tapdancing |  | no | no | 3 |
|  | Twirling | Twirling |  | no | Can be | 3 |
| Equestrian sports |  | Carriage driving | yes | no | Can be | 3 |
|  |  | Dressage |  | no | Can be | 3 |
|  |  | Horse training |  | no | no | 3 |
|  |  | Horseback riding |  | no | Can be | 3 |
| Fighting sports | Boxing | Boxing |  | no | yes | 2 |
|  | Fencing | Fencing |  | no | yes | 2 |
|  | Martial arts | Aiki jitsu |  | no | yes | 2 |
|  |  | Barokai |  | no | yes | 2 |
|  |  | Capoeira |  | no | Can be | 3 |
|  |  | Choi Kwan Do |  | no | yes | 2 |
|  |  | Eskrima |  | no | yes | 2 |
|  |  | Iaido |  | no | yes | 2 |
|  |  | Judo |  | no | yes | 2 |
|  |  | Jujitsu |  | no | yes | 2 |
|  |  | Karate |  | no | yes | 2 |
|  |  | Kempo |  | no | yes | 2 |
|  |  | Kickboxing |  | no | yes | 2 |
|  |  | Kickfun |  | no | yes | 2 |
|  |  | Kobudo |  | no | yes | 2 |
|  |  | Krav Maga |  | no | yes | 2 |
|  |  | Kung Fu |  | no | yes | 2 |
|  |  | Martial arts |  | no | yes | 2 |
| Fighting sports |  | Ninjatsa |  | no | yes | 2 |
| (cont.) |  | Tae kwan do |  | no | yes | 2 |
|  |  | Tang soo do |  | no | yes | 2 |
|  |  | Thai boxing |  | no | yes | 2 |
|  |  | Wing Chun |  | no | yes | 2 |
|  | Sword fighting | Sword fighting |  | no | yes | 2 |
|  | Wrestling | Wrestling |  | no | yes | 2 |
| Fitness training | Bootcamp | Bootcamp |  | no | no | 4 |
|  | Boxing, punching bag | Boxing, punching bag |  | no | no | 4 |
|  | Calisthenics | Calisthenics (abdominal) |  | no | no | 4 |
|  |  | Calisthenics (back exercises) |  | no | no | 4 |
|  |  | Calisthenics (general) |  | no | no | 4 |
|  |  | Curves |  | no | no | 4 |
|  |  | Powerplate |  | no | no | 4 |
|  |  | Slender you |  | no | no | 4 |
|  |  | TMF heat cabine |  | no | no | 4 |
|  | Fitness | 55+ Sport |  | no | no | 4 |
|  |  | Fitness |  | no | no | 4 |
|  |  | Fysiofitness |  | no | no | 4 |
|  |  | Nederlands on the Move |  | no | no | 4 |
|  | Group fitness | 7-min workout |  | no | no | 4 |
|  |  | Body Attack |  | no | no | 4 |
|  |  | Body Boost |  | no | no | 4 |
|  |  | Body Combat |  | no | no | 4 |
|  |  | Body Fit |  | no | no | 4 |
|  |  | Body Five |  | no | no | 4 |
|  |  | Body Heat |  | no | no | 4 |
|  |  | Body Jam |  | no | no | 4 |
|  |  | Body Kick |  | no | no | 4 |
|  |  | Body Power |  | no | no | 4 |
|  |  | Body Pump |  | no | no | 4 |
| Fitness training |  | Body Shape |  | no | no | 4 |
| (cont.) |  | Body Styling |  | no | no | 4 |
|  |  | Bodyline |  | no | no | 4 |
|  |  | Crossfit |  | no | no | 4 |
|  |  | Fat attack/burn |  | no | no | 4 |
|  |  | Femme Fit |  | no | no | 4 |
|  |  | Group fitness |  | no | no | 4 |
|  |  | Keep-fit |  | no | no | 4 |
|  |  | Piloxing |  | no | no | 4 |
|  |  | X-CO |  | no | no | 4 |
|  | Home exercises | Home exercises |  | no | no | 4 |
|  |  | Video workout |  | no | no | 4 |
|  | Pole Fitness | Pole Fitness |  | no | Can be | 4 |
|  | Water aerobics | Water aerobics |  | no | no | 4 |
|  | Weight lifting | Bodybuilding |  | no | yes | 4 |
|  |  | Weight lifting |  | no | Can be | 4 |
|  | Weight training | Circuit training |  | no | no | 4 |
|  |  | CRXworx |  | no | no | 4 |
|  |  | Dry training |  | no | no | 4 |
|  |  | Total body workout |  | no | no | 4 |
|  |  | Weight training (other) |  | no | no | 4 |
| Mind games |  | Bridge | yes | yes | yes | 4 |
|  |  | Checkers | yes | no | yes | 4 |
|  |  | Chess | yes | no | yes | 4 |
|  |  | Mind Games (other) | yes | no | Can be | 4 |
| Motor sports |  | Drag Racing | yes | no | yes | 3 |
|  |  | Go carting | yes | no | yes | 3 |
|  |  | Motor-cross |  | no | yes | 3 |
| Swimming based sports | Diving | Diving |  | no | no | 3 |
|  |  | Scubadiving |  | no | no | 3 |
|  | Lifeguard | Lifeguard |  | no | no | 4 |
| Swimming based sports | Swimming | Competitive swimming |  | no | yes | 4 |
| (cont.) |  | Pregnancy swimming |  | no | no | 4 |
|  |  | Relaxation Swimming |  | no | no | 4 |
|  |  | Rheumatic Swimming |  | no | no | 4 |
|  |  | Swimming |  | no | Can be | 4 |
|  |  | Swimming (laps) |  | no | no | 4 |
|  |  | Swimming (special) |  | no | no | 4 |
|  |  | Swimming (sport) |  | no | Can be | 4 |
|  |  | Swimming (therapeutic) |  | no | no | 4 |
|  |  | Synchronized Swimming |  | no | yes | 3 |
|  | Water polo | Water polo |  | yes | yes | 1 |
| Racket sports | Badminton | Badminton |  | Can be | yes | 2 |
|  |  | Badminton (sitting) |  | Can be | yes | 2 |
|  |  | Competitive badminton |  | Can be | yes | 2 |
|  | Ping pong | Ping pong |  | no | yes | 2 |
|  | Squash | Racquetball |  | no | yes | 2 |
|  |  | Squash |  | no | yes | 2 |
|  | Tennis | Tennis |  | Can be | yes | 2 |
|  |  | Tennis (wheelchair) |  | Can be | yes | 2 |
| Rowing | Canoeing | Canoeing |  | Can be | Can be | 3 |
|  |  | Kayaking |  | Can be | Can be | 3 |
|  | Rowing | Competitive rowing |  | yes | yes | 3 |
|  |  | Rowing |  | yes | Can be | 3 |
| Tai chi/yoga | Pilates | Body Balance |  | no | no | 4 |
|  |  | Pilates |  | no | no | 4 |
|  | Tai chi | Aikido |  | no | no | 3 |
|  |  | Chu Yu Do |  | no | no | 3 |
|  |  | Hapkido |  | no | no | 3 |
|  |  | Pentjak Silat |  | no | no | 3 |
|  |  | Qi gong |  | no | no | 3 |
|  |  | Tai Chi |  | no | no | 3 |
| Thai chi/yoga | Yoga | Baby Balance |  | no | no | 4 |
| (cont.) |  | Ismakogie |  | no | no | 4 |
|  |  | Stretching |  | no | no | 4 |
|  |  | Tacoyo |  | no | no | 4 |
|  |  | Yoga (hatha) |  | no | no | 4 |
|  |  | Yoga (Nadisodhana) |  | no | no | 4 |
|  |  | Yoga (power) |  | no | no | 4 |
|  |  | Yoga (Surya Namaskar) |  | no | no | 4 |
| Track-and-field sports | Jumping | Hurdles |  | no | Can be | 3 |
|  |  | High jump |  | no | Can be | 3 |
|  |  | Fierljeppen (traditional) |  | no | Can be | 3 |
|  |  | Long jump |  | no | Can be | 3 |
|  |  | Pole vault |  | no | Can be | 3 |
|  | Running | Sprinting |  | no | Can be | 4 |
|  | Throwing | Discus Throwing |  | no | yes | 3 |
| Walking based sports | Hiking | Fitwalking |  | no | no | 4 |
|  |  | Hiking |  | no | no | 4 |
|  |  | Mountain hiking |  | no | no | 4 |
|  |  | Marching |  | no | no | 4 |
|  |  | Nordic Walking |  | no | no | 4 |
|  |  | Orienteering |  | no | no | 4 |
|  |  | Survival trail |  | no | Can be | 3 |
|  | Referee/coach | Referee |  | no | no | 4 |
|  |  | Sport coach |  | no | no | 4 |
| Water activities | Fishing | Fishing | yes | no | Can be | 3 |
|  | Jet-skiiing | Jet-skiiing |  | no | no | 3 |
|  |  | Water skiing |  | no | Can be | 4 |
|  | Kiting | Kite flying | yes | no | no | 3 |
|  |  | Kitesurfing |  | no | Can be | 3 |
|  | Sailing | Competitive sailing |  | no | yes | 3 |
|  |  | Sailing |  | no | Can be | 3 |
| Water activities |  | Yachting |  | no | no | 3 |
| (cont.) | Surfing | Flowboarding |  | no | no | 4 |
|  |  | Paddleboarding |  | no | no | 4 |
|  |  | Surfing |  | no | no | 4 |
|  |  | Wakeboarding |  | no | Can be | 4 |
|  |  | Windsurfing |  | no | Can be | 3 |
| Winter activities | Cross Country Skiing | Cross Country Skiing |  | no | Can be | 4 |
|  | Ice Skating | Ice Skating |  | Can be | Can be | 4 |
|  |  | Ice Skating (long track) |  | Can be | yes | 4 |
|  |  | Ice Skating (marathon) |  | Can be | yes | 4 |
|  |  | Ice skating (short track) |  | yes | yes | 4 |
|  | Skiing | Ski-gym |  | no | no | 4 |
|  |  | Skiing (indoor) |  | no | Can be | 4 |
|  |  | Snowboarding (indoor) |  | no | Can be | 4 |

**Supplementary Table I.** This is a complete list of the exercise activities that were reported by the NTR participants (NB: minor variations of the same activity not listed to exhaustion). Domains and subdomains are not used in the analyses and just serve to help the reader navigate this table. Exclude means that the activity was considered below the threshold for moderate-to-vigorous intensity. **Team sports** means any sport that is or can be performed in a team-setting, where multiple individuals work together to achieve a shared objective. If a sport *can be* a team sport is counted as a team sport over a solitary sport in the current paper. **Competitive sport** means a type of sport where a team or individual (can) competes against another team or individual to win. If a sport *can be* a competitive sport is counted as a team sport over a solitary sport in the current paper. **Internal/external pacing** is coded as follows:
1 Highly externally paced, influences from both teammates and opponent
2 Intermediate extenally paced, influenced by opponent or teammates only
3 Low externally paced, influenced by elements (wind/water/ music/synchronic team movements)
4 Internally paced
For the purposes of the current paper, categories 1 and 2 are considered externally paced, and category 4 is considered internally paced. Category 3 is excluded.

**Supplementary table II. Exercise and age descriptives per sex and family relation.**

|  |  | **Age** | **Birth year** | **Total** | **Team** | **Competitive** | **Externally paced** | **Solitary** | **Non-competitive** | **Internally paced** |
| --- | --- | --- | --- | --- | --- | --- | --- | --- | --- | --- |
| Male parents | Mean | 48.21 | 1959 | 673.50 | 137.03 | 244.52 | 234.82 | 536.47 | 428.98 | 414.69 |
|  | SD | 7.41 | 9.95 | 1067.79 | 408.11 | 549.25 | 532.82 | 981.62 | 912.07 | 901.12 |
| Female parents | Mean | 45.89 | 1962 | 502.92 | 39.95 | 118.27 | 116.63 | 462.97 | 384.65 | 345.91 |
|  | SD | 7.68 | 10.10 | 720.78 | 215.11 | 357.05 | 357.31 | 689.37 | 630.01 | 600.38 |
| Male children | Mean | 27.03 | 1978 | 1210.77 | 559.08 | 710.40 | 690.19 | 651.70 | 500.38 | 460.05 |
|  | SD | 12.00 | 12.68 | 1384.59 | 945.37 | 1055.70 | 1044.40 | 1090.23 | 959.64 | 917.58 |
| Female children | Mean | 29.48 | 1977 | 780.26 | 223.30 | 305.69 | 301.97 | 556.96 | 474.57 | 391.08 |
|  | SD | 13.06 | 13.70 | 1022.47 | 627.34 | 707.39 | 711.73 | 854.72 | 783.18 | 712.11 |
| MZM | Mean | 27.26 | 1978 | 1253.26 | 565.40 | 734.03 | 712.84 | 687.86 | 519.22 | 478.73 |
|  | SD | 12.41 | 12.82 | 1421.74 | 928.63 | 1078.47 | 1054.75 | 1146.86 | 970.73 | 918.54 |
| MZF | Mean | 29.88 | 1977 | 796.28 | 229.72 | 325.25 | 318.82 | 566.56 | 471.03 | 392.72 |
|  | SD | 13.63 | 13.96 | 1068.76 | 641.64 | 739.33 | 725.18 | 910.94 | 818.66 | 755.74 |
| DZM | Mean | 24.89 | 1979 | 1258.62 | 646.01 | 788.97 | 769.86 | 612.61 | 469.65 | 421.32 |
|  | SD | 11.17 | 12.22 | 1375.40 | 1039.40 | 1109.98 | 1099.72 | 1027.20 | 922.06 | 891.88 |
| DZF | Mean | 27.20 | 1979 | 824.07 | 254.63 | 332.94 | 330.97 | 569.43 | 491.13 | 399.36 |
|  | SD | 12.00 | 13.07 | 1071.69 | 686.06 | 747.68 | 763.81 | 860.50 | 796.93 | 720.05 |

**Supplementary table II.** MZM: Monozygotic male twins; MZF: Monozygotic female twins; DZM: Males part of a dyzigotic twin pair; DZF: Females part of a dizygotic twin pair; Total: Total volume of exercise (METminutes /week) ; Team: volume of exercise in team-based activities; Comp: volume of exercise in competitive activities; Externally paced: volume of exercise in externally paced activities; Solitary: volume of exercise in solitary activities; Non-competitive: volume of exercise in non-competitive activities; Internally paced: volume of exercise in internally paced activities.

**Supplementary Table III. Estimates from all models tested with the Mendel software package**

|  |  | ***Total*** | | ***Team*** | | ***Solitary*** | | ***Competitive*** | | ***Non-Competitive*** | | ***Ext. Paced*** | | | ***Int. Paced*** | |
| --- | --- | --- | --- | --- | --- | --- | --- | --- | --- | --- | --- | --- | --- | --- | --- | --- |
|  |  | **ACE** | **ACDE** | **ACE** | **ACDE** | **ACE** | **ACDE** | **ACE** | **ACDE** | **ACE** | **ACDE** | **ACE** | **ACDE** | **ACE** | | **ACDE** |
| **Household** | **A** | 0.28 | 0.19 | 0.32 | 0.14 | 0.23 | 0.15 | 0.3 | 0.17 | 0.21 | 0.13 | 0.31 | 0.18 | 0.2 | | 0.13 |
|  | **C** | 0.2 | 0.2 | 0.09 | 0.11 | 0.19 | 0.2 | 0.19 | 0.2 | 0.17 | 0.17 | 0.19 | 0.2 | 0.17 | | 0.17 |
|  | **D** |  | 0.21 |  | 0.32 |  | 0.18 |  | 0.26 |  | 0.17 |  | 0.26 |  | | 0.16 |
|  | **E** | 0.52 | 0.4 | 0.59 | 0.42 | 0.58 | 0.48 | 0.51 | 0.37 | 0.62 | 0.52 | 0.5 | 0.36 | 0.63 | | 0.54 |
|  | ***Log Likelihood*** | *13317* | *13394* | *32470* | *32698* | *11870* | *11925* | *22595* | *22731* | *12312* | *12361* | *23165* | *23308* | *12604* | | *12647* |
| **SibHousehold** | **A** | 0.25 | 0.22 | 0.25 | 0.17 | 0.22 | 0.19 | 0.27 | 0.23 | 0.19 | 0.17 | 0.29 | 0.23 | 0.19 | | 0.16 |
|  | **C** | 0.1 | 0.04 | 0.11 | 0.03 | 0.07 | 0.02 | 0.08 | 0.01 | 0.06 | 0.01 | 0.08 | 0 | 0.05 | | 0 |
|  | **D** |  | 0.15 |  | 0.26 |  | 0.14 |  | 0.21 |  | 0.14 |  | 0.23 |  | | 0.14 |
|  | **E** | 0.65 | 0.59 | 0.64 | 0.53 | 0.72 | 0.65 | 0.64 | 0.55 | 0.74 | 0.68 | 0.63 | 0.54 | 0.76 | | 0.69 |
|  | ***Log Likelihood*** | *12996* | *13018* | *32512* | *32615* | *11574* | *11593* | *22349* | *22407* | *12092* | *12111* | *22907* | *22974* | *12386* | | *12405* |
| **SpouseHousehold** | **A** | 0.34 | 0.26 | 0.36 | 0.19 | 0.28 | 0.22 | 0.36 | 0.26 | 0.25 | 0.19 | 0.37 | 0.26 | 0.24 | | 0.18 |
|  | **C** | 0.25 | 0.24 | 0.19 | 0.16 | 0.25 | 0.25 | 0.26 | 0.25 | 0.22 | 0.22 | 0.27 | 0.26 | 0.21 | | 0.21 |
|  | **D** |  | 0.15 |  | 0.28 |  | 0.12 |  | 0.19 |  | 0.12 |  | 0.19 |  | | 0.12 |
|  | **E** | 0.42 | 0.35 | 0.46 | 0.36 | 0.47 | 0.41 | 0.37 | 0.31 | 0.52 | 0.47 | 0.36 | 0.29 | 0.55 | | 0.49 |
|  | ***Log Likelihood*** | *13411* | *13445* | *32543* | *32709* | *11994* | *12016* | *22720* | *22783* | *12408* | *12431* | *23295* | *23361* | *12676* | | *12697* |
| **TwinHousehold** | **A** | 0.24 | 0.22 | 0.24 | 0.18 | 0.21 | 0.19 | 0.26 | 0.23 | 0.19 | 0.17 | 0.27 | 0.23 | 0.18 | | 0.16 |
|  | **C** | 0.14 | 0.08 | 0.16 | 0.07 | 0.11 | 0.06 | 0.14 | 0.07 | 0.1 | 0.05 | 0.14 | 0.06 | 0.09 | | 0.04 |
|  | **D** |  | 0.12 |  | 0.23 |  | 0.1 |  | 0.16 |  | 0.1 |  | 0.17 |  | | 0.11 |
|  | **E** | 0.61 | 0.58 | 0.6 | 0.53 | 0.68 | 0.65 | 0.6 | 0.55 | 0.71 | 0.68 | 0.59 | 0.54 | 0.73 | | 0.69 |
|  | ***Log Likelihood*** | *13010* | *13022* | *32554* | *32624* | *11590* | *11598* | *22389* | *22417* | *12106* | *12115* | *22949* | *22982* | *12397* | | *12407* |

**Supplementary Table IV. Spousal resemblance and its sources in team-based sports activities.**

| Hypothesis | N | Estimates | Δ-2LL | Δdf | *p* | Spousal Resemblance | Phenotypic Assortment | Social Homogamy | Marital interaction |
| --- | --- | --- | --- | --- | --- | --- | --- | --- | --- |
| *r1* > 0 | 1,608 | 0.24>0 | 61.88 | 1 | 3.66*10^-15^ | √ |  |  |  |
| *r4* > 0 | 10,711 | 0.71>0 | 2335.71 | 1 | < 10^-200^ | √ |  |  |  |
| *r1* > *r2* | 1,420 | 0.24>0.12 | 16.61 | 2 | 2.48*10^-4^ |  | √ | - |  |
| *r2* > *r3* | 419 | 0.12>0.31 | 8.12 | 2 | 0.017 |  | - | √ |  |
| *r2_mz_* > *r2_dz_* | 465 | 0.12>0.12 | 0 | 1 | >0.99 |  | - | √ |  |
| *r3_mz_* > *r3_dz_* | 157 | 0.36>0.14 | 2.02 | 1 | 0.16 |  | - | √ |  |
| *r4* > *r1* | 1,608 | 0.71>0.24 | 656.74 | 1 | 7.66*10^-145^ |  |  |  | √ |

**Supplementary Table IV.** Hypothesis: expectations for the patterns of twin-spouse correlations (r1), co-twin spouse-correlations(r2), spouse1-spouse2 correlations (r3), and parent-parent correlations (r4) under phenotypic assortment, social homogamy, and marital interaction. Under phenotypic assortment the expected pattern of correlations is r1 > r2 > r3, r2MZ > r2DZ , r3MZ > r3DZ. Under social homogamy the expected pattern is (r1 = r2 ≥ r3), r2MZ = r2DZ , r3MZ = r3DZ. Under marital interaction we expect r4 > r1; N: Number of complete pairs in the data (if two correlation coefficients are compared the lowest N is presented); Δ-2LL: Difference in -2 log-likelihood compared to the base model; Δdf: Difference in degrees of freedom compared to the base model, and used in the chi-squared test; p: p-value of the chi-squared difference test comparing the base model to the constrained model; √: Hypothesis supported with α = 0.01.

**Supplementary Table V. Spousal resemblance and its sources in competitive sports activities.**

| Hypothesis | N | Estimates | Δ-2LL | Δdf | *p* | Spousal Resemblance | Phenotypic Assortment | Social Homogamy | Marital interaction |
| --- | --- | --- | --- | --- | --- | --- | --- | --- | --- |
| *r1* > 0 | 1,608 | 0.23>0 | 81.91 | 1 | 1.43*10^-19^ | √ |  |  |  |
| *r4* > 0 | 10,711 | 0.6>0 | 1525.94 | 1 | < 10^-200^ | √ |  |  |  |
| *r1* > *r2* | 1,420 | 0.23>0.07 | 36.25 | 2 | 1.35*10^-8^ |  | √ | - |  |
| *r2* > *r3* | 419 | 0.07>0.2 | 4.16 | 2 | 0.12 |  | - | √ |  |
| *r2_mz_* > *r2_dz_* | 465 | 0.06>0.11 | 1.47 | 1 | 0.22 |  | - | √ |  |
| *r3_mz_* > *r3_dz_* | 157 | 0.18>0.23 | 0.19 | 1 | 0.66 |  | - | √ |  |
| *r4* > *r1* | 1,608 | 0.6>0.23 | 326.41 | 1 | 5.80*10^-73^ |  |  |  | √ |

**Supplementary Table V.** Hypothesis: expectations for the patterns of twin-spouse correlations (r1), co-twin spouse-correlations(r2), spouse1-spouse2 correlations (r3), and parent-parent correlations (r4) under phenotypic assortment, social homogamy, and marital interaction. Under phenotypic assortment the expected pattern of correlations is r1 > r2 > r3, r2MZ > r2DZ , r3MZ > r3DZ. Under social homogamy the expected pattern is (r1 = r2 ≥ r3), r2MZ = r2DZ , r3MZ = r3DZ. Under marital interaction we expect r4 > r1; N: Number of complete pairs in the data (if two correlation coefficients are compared the lowest N is presented); Δ-2LL: Difference in -2 log-likelihood compared to the base model; Δdf: Difference in degrees of freedom compared to the base model, and used in the chi-squared test; p: p-value of the chi-squared difference test comparing the base model to the constrained model; √: Hypothesis supported with α = 0.01.

**Supplementary Table VI. Spousal resemblance and its sources in externally paced sports activities.**

| Hypothesis | N | Estimates | Δ-2LL | Δdf | *p* | Spousal Resemblance | Phenotypic Assortment | Social Homogamy | Marital interaction |
| --- | --- | --- | --- | --- | --- | --- | --- | --- | --- |
| *r1* > 0 | 1,608 | 0.26>0 | 93.04 | 1 | 5.12*10^-22^ | √ |  |  |  |
| *r4* > 0 | 10,711 | 0.61>0 | 1664.68 | 1 | < 10^-200^ | √ |  |  |  |
| *r1* > *r2* | 1,420 | 0.26>0.11 | 31.94 | 2 | 1.16*10^-7^ |  | √ | - |  |
| *r2* > *r3* | 419 | 0.11>0.2 | 2.58 | 2 | 0.28 |  | - | √ |  |
| *r2_mz_* > *r2_dz_* | 465 | 0.1>0.13 | 0.37 | 1 | 0.54 |  | - | √ |  |
| *r3_mz_* > *r3_dz_* | 157 | 0.17>0.24 | 0.4 | 1 | 0.53 |  | - | √ |  |
| *r4* > *r1* | 1,608 | 0.61>0.26 | 314.86 | 1 | 1.91*10^-70^ |  |  |  | √ |

**Supplementary Table VI.** Hypothesis: expectations for the patterns of twin-spouse correlations (r1), co-twin spouse-correlations(r2), spouse1-spouse2 correlations (r3), and parent-parent correlations (r4) under phenotypic assortment, social homogamy, and marital interaction. Under phenotypic assortment the expected pattern of correlations is r1 > r2 > r3, r2MZ > r2DZ , r3MZ > r3DZ. Under social homogamy the expected pattern is (r1 = r2 ≥ r3), r2MZ = r2DZ , r3MZ = r3DZ. Under marital interaction we expect r4 > r1; N: Number of complete pairs in the data (if two correlation coefficients are compared the lowest N is presented); Δ-2LL: Difference in -2 log-likelihood compared to the base model; Δdf: Difference in degrees of freedom compared to the base model, and used in the chi-squared test; p: p-value of the chi-squared difference test comparing the base model to the constrained model; √: Hypothesis supported with α = 0.01.

**Supplementary Table VII. Spousal resemblance and its sources in solitary paced sports activities.**

| Hypothesis | N | Estimates | Δ-2LL | Δdf | *p* | Spousal Resemblance | Phenotypic Assortment | Social Homogamy | Marital interaction |
| --- | --- | --- | --- | --- | --- | --- | --- | --- | --- |
| *r1* > 0 | 1,608 | 0.16>0 | 69.15 | 1 | 9.12*10^-17^ | √ |  |  |  |
| *r4* > 0 | 10,711 | 0.28>0 | 692.13 | 1 | 1.54*10^-152^ | √ |  |  |  |
| *r1* > *r2* | 1,420 | 0.16>0.08 | 18.55 | 2 | 9.37*10^-5^ |  | √ | - |  |
| *r2* > *r3* | 419 | 0.08>0.06 | 0.71 | 2 | 0.70 |  | - | √ |  |
| *r2_mz_* > *r2_dz_* | 465 | 0.07>0.1 | 0.34 | 1 | 0.56 |  | - | √ |  |
| *r3_mz_* > *r3_dz_* | 157 | 0.07>0.03 | 0.18 | 1 | 0.67 |  | - | √ |  |
| *r4* > *r1* | 1,608 | 0.28>0.16 | 27.8 | 1 | 1.35*10^-7^ |  |  |  | √ |

**Supplementary Table VII.** Hypothesis: expectations for the patterns of twin-spouse correlations (r1), co-twin spouse-correlations(r2), spouse1-spouse2 correlations (r3), and parent-parent correlations (r4) under phenotypic assortment, social homogamy, and marital interaction. Under phenotypic assortment the expected pattern of correlations is r1 > r2 > r3, r2MZ > r2DZ , r3MZ > r3DZ. Under social homogamy the expected pattern is (r1 = r2 ≥ r3), r2MZ = r2DZ , r3MZ = r3DZ. Under marital interaction we expect r4 > r1; N: Number of complete pairs in the data (if two correlation coefficients are compared the lowest N is presented); Δ-2LL: Difference in -2 log-likelihood compared to the base model; Δdf: Difference in degrees of freedom compared to the base model, and used in the chi-squared test; p: p-value of the chi-squared difference test comparing the base model to the constrained model; √: Hypothesis supported with α = 0.01.

**Supplementary Table VIII. Spousal resemblance and its sources in non-competitive sports activities.**

| Hypothesis | N | Estimates | Δ-2LL | Δdf | *p* | Spousal Resemblance | Phenotypic Assortment | Social Homogamy | Marital interaction |
| --- | --- | --- | --- | --- | --- | --- | --- | --- | --- |
| *r1* > 0 | 1,608 | 0.14>0 | 47.22 | 1 | 6.33*10^-12^ | √ |  |  |  |
| *r4* > 0 | 10,711 | 0.23>0 | 535.41 | 1 | 1.88*10^-118^ | √ |  |  |  |
| *r1* > *r2* | 1,420 | 0.14>0.06 | 13.19 | 2 | 1.37*10^-3^ |  | √ | - |  |
| *r2* > *r3* | 419 | 0.06>0.07 | 0.61 | 2 | 0.74 |  | - | √ |  |
| *r2_mz_* > *r2_dz_* | 465 | 0.06>0.09 | 0.43 | 1 | 0.51 |  | - | √ |  |
| *r3_mz_* > *r3_dz_* | 157 | 0.08>0.03 | 0.25 | 1 | 0.61 |  | - | √ |  |
| *r4* > *r1* | 1,608 | 0.23>0.14 | 19.98 | 1 | 7.82*10^-6^ |  |  |  | √ |

**Supplementary Table VIII.** Hypothesis: expectations for the patterns of twin-spouse correlations (r1), co-twin spouse-correlations(r2), spouse1-spouse2 correlations (r3), and parent-parent correlations (r4) under phenotypic assortment, social homogamy, and marital interaction. Under phenotypic assortment the expected pattern of correlations is r1 > r2 > r3, r2MZ > r2DZ , r3MZ > r3DZ. Under social homogamy the expected pattern is (r1 = r2 ≥ r3), r2MZ = r2DZ , r3MZ = r3DZ. Under marital interaction we expect r4 > r1; N: Number of complete pairs in the data (if two correlation coefficients are compared the lowest N is presented); Δ-2LL: Difference in -2 log-likelihood compared to the base model; Δdf: Difference in degrees of freedom compared to the base model, and used in the chi-squared test; p: p-value of the chi-squared difference test comparing the base model to the constrained model; √: Hypothesis supported with α = 0.01.

**Supplementary Table IX. Spousal resemblance and its sources in internally paced sports activities.**

| Hypothesis | N | Estimates | Δ-2LL | Δdf | *p* | Spousal Resemblance | Phenotypic Assortment | Social Homogamy | Marital interaction |
| --- | --- | --- | --- | --- | --- | --- | --- | --- | --- |
| *r1* > 0 | 1,608 | 0.14>0 | 48.93 | 1 | 2.66*10^-12^ | √ |  |  |  |
| *r4* > 0 | 10,711 | 0.22>0 | 496.66 | 1 | 5.06*10^-110^ | √ |  |  |  |
| *r1* > *r2* | 1,420 | 0.14>0.07 | 15.32 | 2 | 4.71*10^-4^ |  | √ | - |  |
| *r2* > *r3* | 419 | 0.07>0.06 | 0.89 | 2 | 0.64 |  | - | √ |  |
| *r2_mz_* > *r2_dz_* | 465 | 0.05>0.1 | 1.1 | 1 | 0.30 |  | - | √ |  |
| *r3_mz_* > *r3_dz_* | 157 | 0.08>0.03 | 0.23 | 1 | 0.63 |  | - | √ |  |
| *r4* > *r1* | 1,608 | 0.22>0.14 | 12.1 | 1 | 5.04*10^-4^ |  |  |  | √ |

**Supplementary Table IX.** Hypothesis: expectations for the patterns of twin-spouse correlations (r1), co-twin spouse-correlations(r2), spouse1-spouse2 correlations (r3), and parent-parent correlations (r4) under phenotypic assortment, social homogamy, and marital interaction. Under phenotypic assortment the expected pattern of correlations is r1 > r2 > r3, r2MZ > r2DZ , r3MZ > r3DZ. Under social homogamy the expected pattern is (r1 = r2 ≥ r3), r2MZ = r2DZ , r3MZ = r3DZ. Under marital interaction we expect r4 > r1; N: Number of complete pairs in the data (if two correlation coefficients are compared the lowest N is presented); Δ-2LL: Difference in -2 log-likelihood compared to the base model; Δdf: Difference in degrees of freedom compared to the base model, and used in the chi-squared test; p: p-value of the chi-squared difference test comparing the base model to the constrained model; √: Hypothesis supported with α = 0.01.

**Figure Captions**

**Supplementary Figure 1. ACDE estimates for other household definitions:** Proportion of variance explained by **additive genetic (A)**, **non-additive genetic (D)**, **common household environmental (C)**, **unique environmental (E)** factors and broad sense heritability(*****) for different classes of exercise using various household definitions as shared environment.
